# Population dynamics of the critically endangered toad *Atelopus cruciger* and the fungal disease chytridiomycosis

**DOI:** 10.1371/journal.pone.0179007

**Published:** 2017-06-01

**Authors:** Margarita Lampo, Celsa Señaris, Carmen Zulay García

**Affiliations:** Centro de Ecología, Instituto Venezolano de Investigaciones Científicas, Kilómetro 11 Carretera Panamericana, Caracas, Venezuela; National Zoological Park, UNITED STATES

## Abstract

Harlequin toads (*Atelopus*) are among the most severely impacted amphibians by the emergence of chytridiomycosis, a fungal disease caused by the pathogen *Batrachochytrium dendrobatidis* (*Bd*). Many species disappeared while others suffered drastic contractions of their geographic distribution to lower altitudes. A diminished virulence of *Bd* in warm habitats was proposed to explain the survival of lowland populations of harlequin toads (*i.e.* thermal refuge hypothesis). To understand the mechanisms that allow some populations to reach an endemic equilibrium with this pathogen, we estimated demographic and epidemiological parameters at one remnant population of *Atelopus cruciger* in Venezuela using mark-recapture data from 2007–2013. We demonstrated that *Bd* is highly virulent for *A. cruciger*, increasing the odds of dying of infected adults four times in relation to uninfected ones and reducing the life expectancy of reproductive toads to a few weeks. Despite an estimated annual loss of 18% of the reproductive population due to *Bd*-induced mortality, this population has persisted in an endemic equilibrium for the last decade through the large recruitment of healthy adults every year. Given the high vulnerability of harlequin toads to *Bd* in lowland populations, thermal refuges need to be redefined as habitats of reduced transmission rather than attenuated virulence.

## Introduction

Chytridiomycosis is an emerging disease that has been linked to population declines and local extinction of more than 200 amphibian species [1]. The pathogen that causes it, *Batrachochytrium dendrobatidis* (*Bd*), is a chytrid fungus that invades the skin of adult amphibians [2, 3]. *Bd* has been detected in, at least, 520 amphibian species and on every continent where amphibians are found [4]. Although the pathogenesis of the disease is not entirely understood, disruption of epidermal function, critical for the maintenance of homeostasis in post-metamorphic individuals, appears to be the cause of morbidity and mortality [5].

This fungal disease has been implicated in the disappearance of several species of harlequin toads (*Atelopus*), a Neotropical genus with 85 of its 97 described species critically endangered and three extinct [6]. Massive die-offs in *Atelopus chiriquiensis* and *A. varius* were observed in Central America during epidemic outbreaks of this disease in 1994 and 1997 [2]. Many other *Atelopus* species suffered drastic declines during the last two decades [7–10] and at least 40 species have not been sighted since the early nineties, and may even be extinct. Harlequin toads are particularly vulnerable to extinction because most species have small geographic ranges [11], although some species with large distributions have also been affected [12–14].

Despite its devastating effect on harlequin frogs in the past, *Bd* persists in some populations of harlequin toads that show no signs of decline [15–17]. Five lowland species [*Atelopus cruciger*, *A. flavescens*, *A. hoogmoedi*, *A. gracilis* (= *Atelopus elegans gracilis*, and *A. pulcher*)] and one highland species, [*A. laetissimus*] appear to maintain populations with abundant individuals despite the presence of *Bd* [16–21]. One common denominator in most of these populations is their occurrence in lowland habitats [12, 15]. Some authors claim that these habitats constitute “thermal refuges” where *Bd* has an attenuated virulence (*e.g.* case mortality rate) because it tends grow slowly at higher temperatures [22–25]. Others have associated the ability of some populations of *Atelopus elegans* to persist with *Bd* with the presence of anti-fungal bacteria [15]. However, no assessments have been made on the impact of this disease on the few remnant populations of harlequin toads and estimates of *Bd*-induced mortality are not available for wild populations.

*Atelopus cruciger* is one the few harlequin toad species that reappeared in the last ten years. Two nearby populations were detected between 2004 and 2006 at low-altitude localities in the northern slope of the Coastal Cordillera in Venezuela. No other specimens have been sighted, despite several explorations of its historic localities [12]. A previous mark-recapture study using a Jolly-Seber-Cormack model suggested that adults from one of these populations have high mortality rates, but high recruitment rates compensate for these losses [17]. The fraction of this mortality attributed to *Bd* infection, however, is not known.

Understanding the mechanisms by which some amphibian species persist with endemic infections is crucial to inform management decisions for its long-term conservation, but quantifying disease impact and pathogen transmission in wildlife populations remains a challenge. The development of Multi-State Capture-Recapture Models (MSCRM) opened new avenues for epidemiological studies in free-ranging wildlife populations, by allowing the modeling of transitions between disease states (*e.g.* infected vs. uninfected) and the maximum likelihood estimation of state-specific survivals or capture probabilities [26, 27]. Here we used capture-recapture data to study the population dynamics and estimate demographic and epidemiological parameters in one remnant population of a critically endangered toad infected with *Bd*.

## Materials and methods

### Individual identification and *Bd* diagnosis

Thirty-five mark-recapture sessions were carried out between April 2007 and February 2013 in a 250 x 3 m transect along a section of the Cata River (Aragua State) on the northern slope of the Cordillera de la Costa, Venezuela. The study site was described earlier [17]. The study was approved by the Comisión Interministerial para el Acceso a Recursos Genéticos del Ministerio del Poder Popular para el Ambiente (Venezuela), a governmental office that grants access to genetic resources and evaluates bioethical issues related with the use of wildlife (Permits No. 01-03-03-3644, No. 4110 to JCS, No. 3502 and No. 3506 to F. Nava and ML, and No. 2573 to F. Nava). *Atelopus cruciger* is currently listed by the International Union for the Conservation of Nature (IUCN) in the Critically Endangered category. The study did not involve the collection of specimens or any invasive sampling. All adults and juveniles sighted were photographed, sexed and their snout-vent lengths (SVL) measured. Sex was determined by the shape of the thumb (long in females vs. short and broad in males), the presence of brownish horny pad at the base of the thumb (only in males), the presence of eggs within the body cavity (females), or their behavior (calling males; pairs in amplexus). All individuals smaller than 20 mm SVL were classified as juveniles [28]. Because of their unique and invariant dorsal color pattern, adults of *A. cruciger* can be unequivocally identified. A number code was assigned to each toad, and a photographic catalog was assembled and updated after each session.

To test toads for *Bd* infection, skin samples were collected from each individual using sterile swabs [29]. Toads were immediately released where they were captured, and the swabs were stored at ambient temperature until arrival to the lab the next day. In the lab, swabs were kept at 4°C until processed within three days after samples were collected. Nucleic acids were extracted from swabs with PrepMan Ultra and *Bd* infection was diagnosed and zoospore loads estimated using real-time PCR Taqman assays in a Opticon thermocycler [29]. Negative controls were used during the extraction procedure to detect DNA contamination. Calibration curves were constructed by including internal 100, 10, 1 and 0.1 *Bd* zoospore quantification standards in each assay (provided by A. Hyatt, Australian Animal Health Laboratory-AAHL, Division of Livestock Industries, CSIRO, Victoria, Australia). Each sample was run in triplicate [30] and the mean number of zoospores for each swab was estimated by extrapolating the *C*_t_ values to their genome equivalents in the calibration curves and correcting for dilution between extraction and quantification. If one replicate differed significantly from the other two, the sample was analyzed again.

### Mark-recapture models

Live encounter histories were constructed for each individual based on whether they were seen and tested positive (*I*) or negative (*U*) for *Bd*, or they were not-seen (*0*) during each sampling session. We excluded juveniles because their dorsal pattern changed with time and therefore, did not allow for the unequivocal identification of individuals. Encounter histories were analyzed using two mark-recapture formulations: a POPAN formulation of the Jolly-Seber model (POPAN JS) [31] to estimate demographic parameters (*i.e.* recruitment, survival and abundance) and a Multi-strata formulation of the Jolly-Move model (MSMRM) to estimate epidemiological parameters (*i.e.* infection rates, the probability of clearing infection and the disease-induced mortality of adults) [32]. For the POPAN JS, toads that tested positive or negative were not distinguished but coded as seen (*1*). Because time intervals between sessions varied and unequal sampling intervals may lead to biased estimates of state-transitions in the MSMRM models, months for which we had no sampling session were coded with dots, indicating “no data available” for these models [33]. To explore seasonal effects on population parameters, we used an environmental covariate that indicated whether the sampling session occurred during the dry or the wet season. For both types of models, we used the ‘RMark’ package v.2.1.9 [34] in R as a front interface to specify models for mark-recapture data analysis in the program Mark v.6.1 [35].

To evaluate the underlying assumptions of the POPAN JS model or the MSMRM model, we tested the data against the correspondent fully time-dependent models by goodness of fit (GOF) tests. For the POPAN model, we used the package release.gof implemented in ‘RMark’ [34] and for the MSMRM the JollyMove model implemented in program U-CARE v.2.3.2 [36, 37]. In both cases, *χ*^2^- or *G*^2^-tests were used to detect transience of newly marked individuals [38], and trap-dependence of recapture rates [36]. The variance inflation factor, *ĉ*, indicating the degree of overdispersion of the data or lack of fit of these general models were estimated as the quotient between the *χ*^2^ and *df* [36].

#### The demographic model

Encounter histories from 542 toads from 35 sampling sessions were used to test the demographic models (S1 Dataset). We constructed 36 alternative models to test hypotheses about sex- or season-dependence of apparent survival, Φ, capture, *p*, and entrance (recruitment) probabilities, *b*. Permanent emigration is indistinguishable from mortality and the combination of both are treated as mortality. Similarly, *in situ* natural births and immigration from outside the study area cannot be separated and, thus, both are treated as births. *Logit Link* functions were used for *p*, and for Φ, and the *Log Link* function for the super-population size, N*, as the latter is not constrained (-∞, ∞). Multinomial *Logit Link* functions were used for *b* as the sum of all entrance probabilities must sum to one within each group. Models were contrasted using the small sample Akaike’s Information Criterion (c*AIC*) after adjusting the *ĉ* values for overdispersion (_Q_ *AIC*), if necessary. The ‘best’ model was the less parameterized model that differed from the model with the lowest _Q_*AIC*_C_ in less than three units [39]. The MLE estimates for the ‘best demographic’ model were used to construct 95% CI for the estimated *p*, Φ, and *b*. The net number of new entrants, *B*, the abundances, *N*, and the super-population size, N*, were derived from these MLE and their standard errors were calculated using the Delta method [31]. Non-estimable parameters were identified using a threshold value of the vector of conditioned singular values of the Hessian matrix, which result from the singular value decomposition of the Hessian divided by the maximum value in this vector [40].

#### The epidemiological model

We used capture histories from 409 adults (265 males and 144 females), obtained during 31 sessions to test epidemiological models because we had no skin samples for four sessions (S2 Dataset). Thirty-six alternative models were constructed to test hypotheses about i) status-dependence of survival and capture probabilities, ii) year-dependence of survival and iii) sex-dependence of survival and capture probabilities. We initially included infection rate among the parameters to be estimated by the models but, due to the large number of equally supported models obtained, we fixed the monthly infection rate to the force of infection λ estimated from the size vs. prevalence curve [41], after size was converted to age using a post-metamorphic growth curve (see next section). Models were contrasted using the small sample Akaike’s Information Criterion (_c_ *AIC*) as described above for the demographic models. MLE were obtained for the survivals, Φ, the capture probabilities, *p* and for the transition probability from infected to uninfected (*i.e*. infection clearance), Ψ^IU^. *Logit Link* functions were used for estimating survivals and capture probabilities and *Multinomial Logit Link* functions for the infection clearance probability. Non-estimable parameters were identified as described for the demographic models.

### Post-metamorphic growth and age-structure

Because it is not possible to age toads in the field, we have no direct estimates of the age structure of the population under study. Size increments over short periods (*i.e.* periodic increment data), however, can be use to reconstruct growth curves from which the age structure of the population may be indirectly inferred [42]. To reconstruct growth curves for post-metamorphic toads, we used a von Bertalanffy growth curve described by the following equation,

$$\begin{array}{c} S=S_{max}\left(1-e^{-K(A-A_{0})}\right) \end{array}$$ (1)

where *S* is the size of toads, *S_max_* is the maximum size of toads, *A* is the age of post-metamoprphic toads, *A_o_* is an age correction factor to set the minimum size of metamorphs to 5 mm and *K* is the rate at which growth rate declines with size. We could only reconstruct growth curves for males due to insufficient numbers of recaptured females. We regressed the snout-vent length of each recaptured toad versus the time between recaptures to obtain their growth rates, *dS*/*dt*. We then regressed the growth rate *dS*/*dt* for each recaptured toad versus its snout-vent length to obtain *K* and *S_max_* (the slope and *X*-intercept of the *dS*/*dt* vs. size regression, respectively). *K* and *S_max_* were then used in Eq 1. to obtain the size-age relationship, from which the age of post-metamorphic toads was estimated.

### Host size and season related changes in prevalence

The prevalence of infection was calculated as the fraction of toads infected with *Bd* at a particular sampling session. We explored whether the prevalence of infection was constant over time or, on the contrary, had a maximum value by fitting the prevalence vs. month relationship to a first-order polynomial (straight line) and second-order polynomial (humped-shaped function) and tested the significance of the decrease in deviance that results from the addition of the non-linear term by an *F*-test [43]. Also, the relationship between the prevalence of infection and the toad SVL (as a proxy of age) was determined to estimate the *per capita* rate at which toads acquire infection (*e.g.* force of infection) and the size at which individuals typically acquire infection [44]. If hosts are always susceptible or their susceptibility increases with age, we expect the prevalence of infection to increase towards a maximum of 100% infected toads. However, any convexity or asymptote in the age-prevalence curve indicates a decrease in the susceptibility of toads with age, the clearing of infection or the loss of infected toads from the population. We used SVL of toads as indicative of their age. Toads were assigned to size classes and the prevalence of infection for each size-class was estimated as the fraction of infected individuals. To explore if the prevalence of infection, *X*_S_, increased monotonically with size until all population is infected, or on the contrary, it reached an asymptotic prevalence less than one, we fitted the fraction of infected toads in each class to the following function using a Nelder-Mead simplex algorithm implemented in Matlab to maximize the likelihood [45]:

$$\begin{array}{c} X_{S}=A*\left(1-e^{-\gamma (S-S_{0})}\right) \end{array}$$ (2)

where *A* = λ/(λ+*ρ*) is the asymptotic proportion of positive toads, λ is the force of infection, *ρ* is the rate of substitution of infected by uninfected toads due to ridding infection or the death of infected toads, *γ* = λ+*ρ*, *S* is the size of toads and *S*_o_ is the threshold size above which toads become susceptible to infection [45]. Maximum likelihood estimators of *A*, *γ* and *S*_o_ were obtained from the fitting Eq 2. to the data and λ was estimated as the product *Aγ*. A value of *A* significantly less than one indicates an asymptote in the age-prevalence curve.

## Results

**Fig 1:**
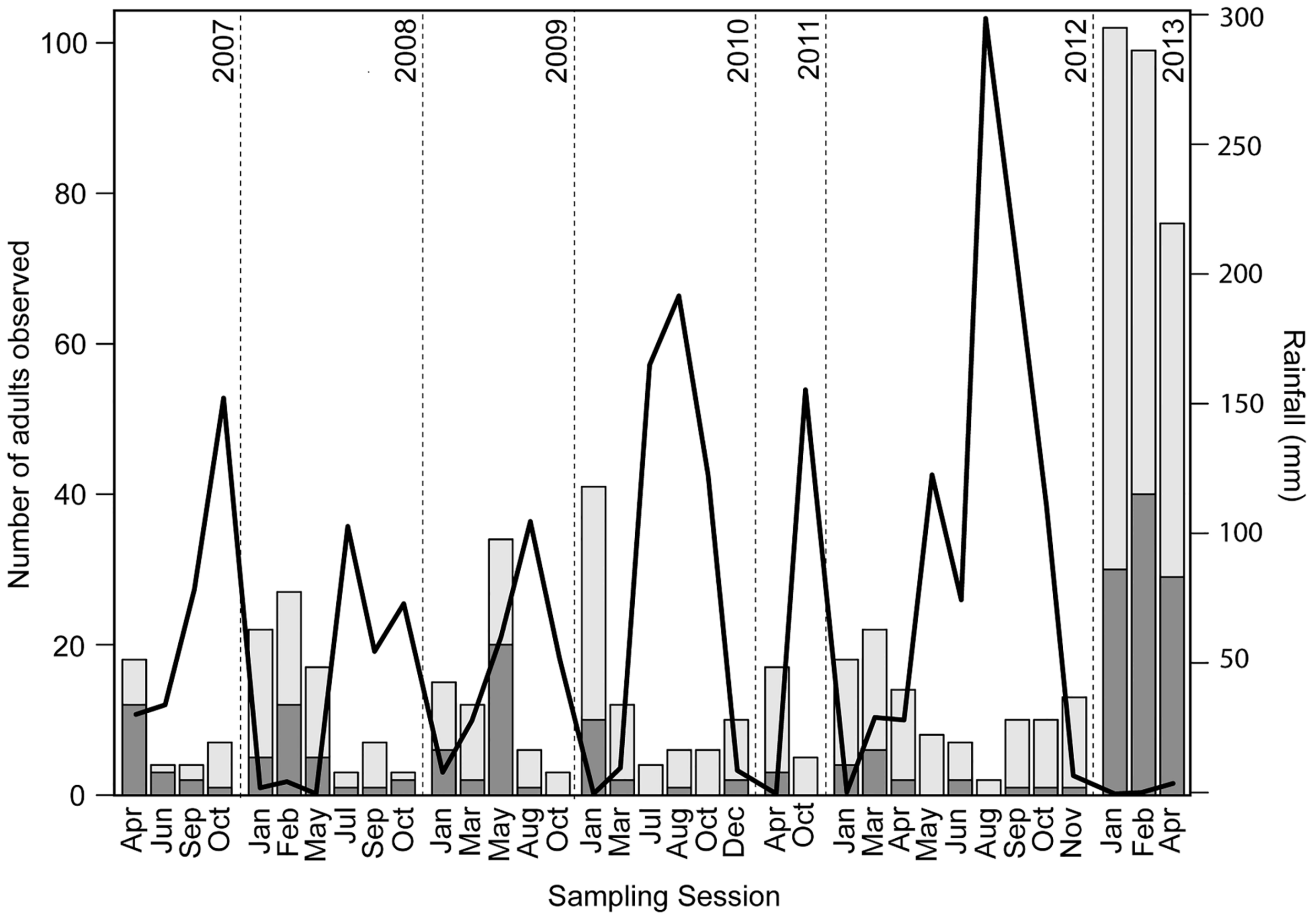
Captures. Number of males (dark grey) and females (light grey) harlequin toads *A. cruciger* captured in each sampling session along a 250 x 3 m transect at Río Cata, Edo. Aragua, Venezuela. The line corresponds to the monthly rainfall from weather stations at Ocumare De La Costa (2007–2011) and Maracay (2012–2013) (S3 Dataset).

A total of 542 toads were identified in 2007–2013. Three-hundred-nine toads (57.0%) were males, 176 (32.4%) females and 57 (10.5%) could not be sexed. The number of captures within the transect varied substantially between sampling sessions with a minimum of ten individuals in August 2012 (wet season) and a maximum of 105 in February 2013 (dry season) (Fig 1).

### Population size and age structure

**Fig 2:**
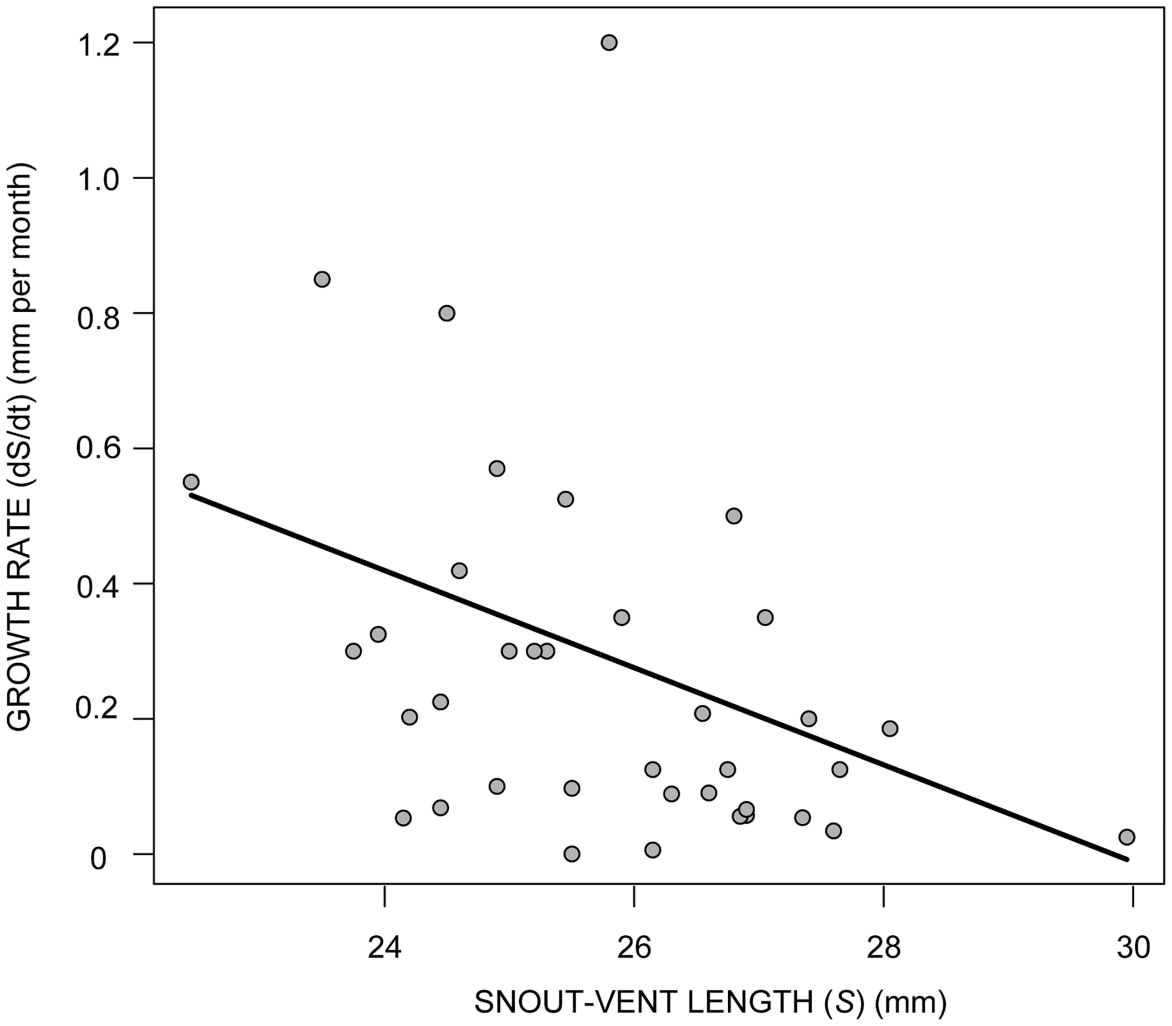
Post-metamorphic rate of growth. Size related changes in the growth rate *dS/dt* of post-metamorphic males of *A. cruciger*. The line corresponds to the fitted model *dS/dt* = *C*-K*S with *C* = 2.143 and *K* = 0.0718.

**Fig 3:**
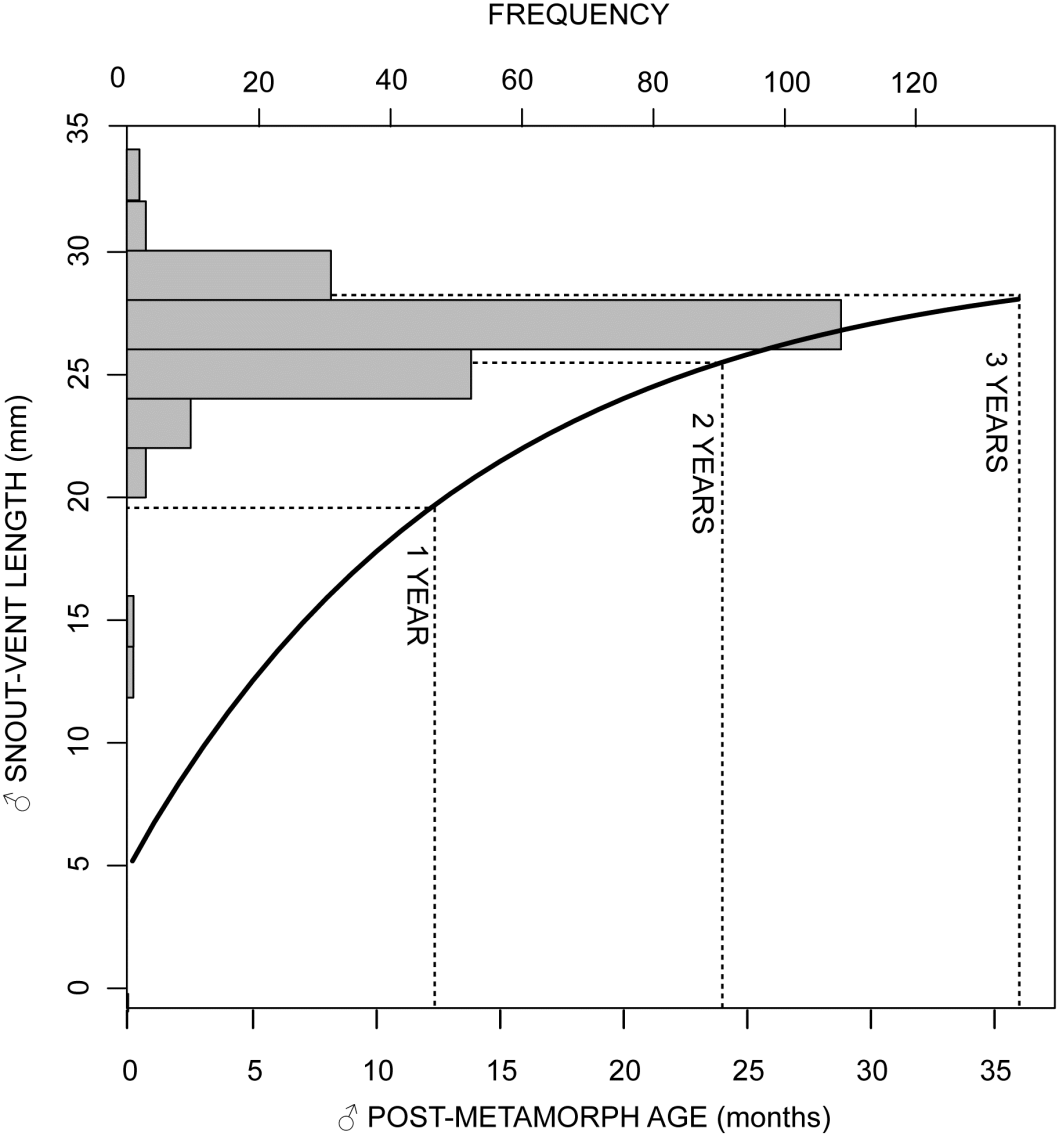
Growth curve of post-metamorphic toads. A von Bertalanffy growth (Eq 2) resulting from the relationship between the growth rate and the size of post-metamorphic male toads (see Fig 2) with *K* = 0.08139 and *S_max_* = 27.9mm. The histogram describes the frequency distribution of sizes of male toads. The dotted lines indicate the predicted ages of the captured males.

The size distribution of toads indicated that 80% of the captured individuals had SVLs between 24 and 32 mm, 9.2% were under 24 mm and 10.8% over 34 mm. With the exception of recently metamorphosed toads (5 mm) observed in few occasions, the smallest toad ever captured was a 12 mm juvenile and the largest was a female of 41.4 mm of length. Females tend to be larger than males. The mean snout-vent length was 31.9 mm for females and 26.6 mm for males. The *dS/dt* vs. size regression showed that the rate of growth of males significant decreased with size (*F* = 5.12; *df* = 1, 36; *p* = 0.0298) (Fig 2). The slope and the *X*-intercept of the fitted linear function suggests that the growth rate of males decreases with size at a rate of 0.0718 mm per month, until males attain an average maximum size of approximately 30 mm. Their reconstructed growth curve and their size distribution indicate that most males captured had between two and three years of age and, only occasionally, we observed males under two years of age (Fig 3). During their first two years, most post-metamorphic and juvenile toads hide away from the water and, therefore, are not susceptible to be captured along the river shore. Only after then, when they are most likely to be reproductively active, toads move near the water to seek for mates and become susceptible to be captured. Thus, parameters estimated by the demographic and epidemiological models correspond to the reproductive population. The small number of recaptured females did not allow for the reconstruction of their growth rate curve or the estimation of their ages.

### Abundance and recruitment

The GOF of the saturated (all time-dependent parameters) demographic model failed to detect any serious evidence of transience of newly marked individuals (*χ*^2^ = 19.5; *df* = 32; *p* = 0.959) or trap-dependence of recaptured toads (*χ*^2^ = 22.4; *df* = 45; *p* = 0.999). The variance inflation factor was estimated to be 0.54 indicating a slight underdispersion of data, possibly as an artifact of the sparseness of data. As *ĉ*<1, no correction was made for the inflation factor [46]. The four top-ranking demographic models are shown in Table 1. In all of these models, time was a better predictor of recruitment than season. In two models, sex was the solely predictor of adult apparent survival, but it was combined with season in the other two models. Conversely, season was the solely predictor of capture probabilities in two models but it was combined with sex in the others. Because these four models differed little in their Akaike values (<3 units), we chose the model with the lowest number of parameters (Φ[sex], *p*[season], *pent*[time]) as the ‘best demographic model’. This model estimated 39 parameters; two survival probabilities, two capture probabilities and 35 recruitment probabilities. However, 11 recruitment probabilities were identified as non-estimable by the threshold conditioned *S*-vector and thus were omitted from the results.

**Table 1:** Model ranking based on a small sample Akaike Information Criteria of Cormack-Jolly-Seber models formulated with POPAN using RMark.

Models	Parameters	AICc	Delta AICc	Weight	Deviance
Φ(sex), *p*(season, sex), *pent*(time)	40	1,455.6	0	0.391	-1991.3
Φ(sex), *p*(season), *pent*(time)	39	1,455.7	0.171	0.359	-1988.9
Φ(season, sex), *p*(season, sex), *pent*(time)	41	1,457.8	2.206	0.129	-1991.4
Φ(season, sex), *p*(season)*pent*(time)	40	1,457.9	2.376	0.119	-1988.9
Φ(.), *p*(season, sex)*pent*(time)	39	1,467.3	11.724	0.001	-1977.3

Only the five top-ranking models are shown. For all others the model weight< 0.001.

**Fig 4:**
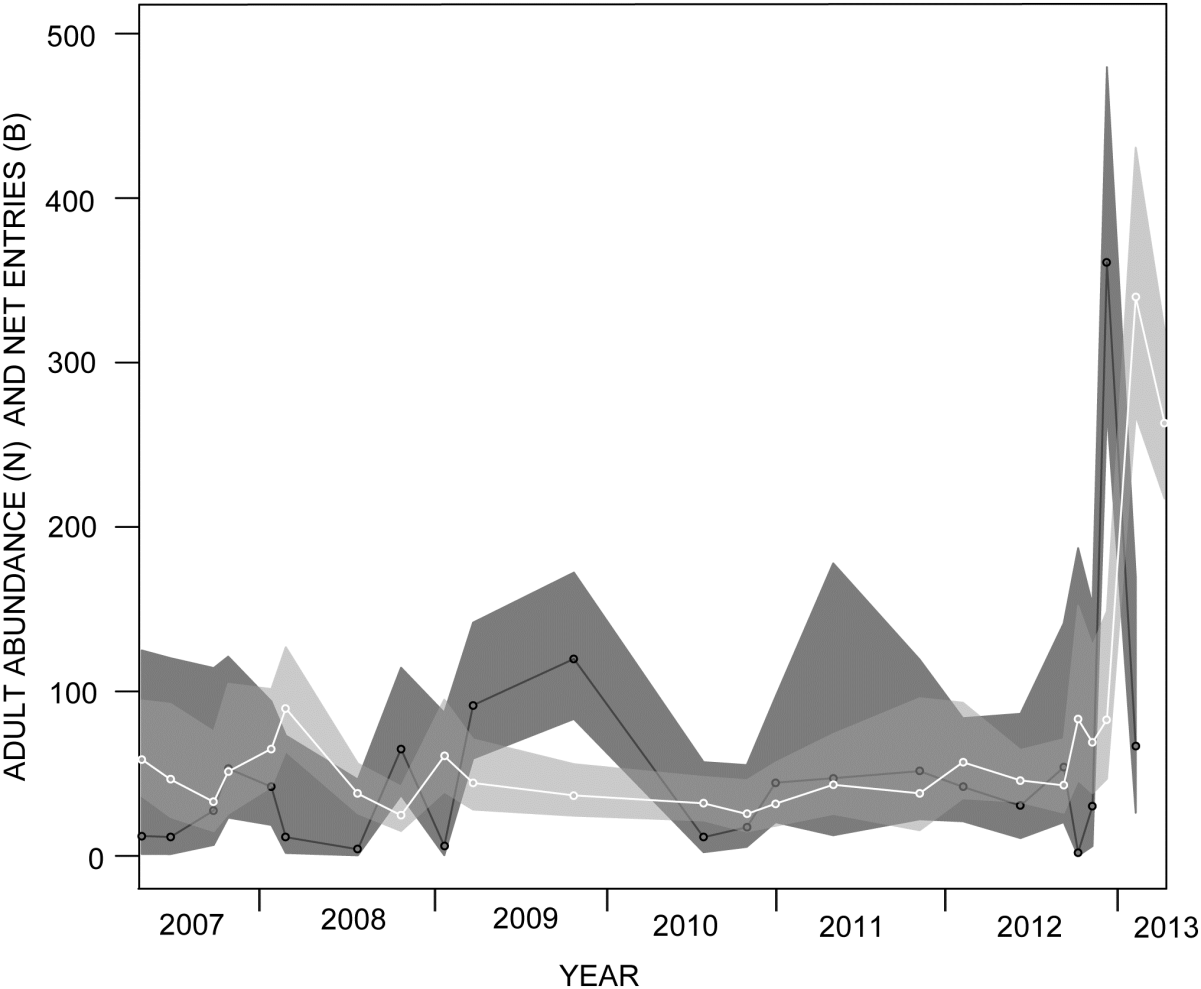
Adult abundance and recruitment. Maximum likelihood estimates (lines) and 95% confidence intervals (bands) for the abundance (white and light grey) and recruitment (black and dark grey) of adults from a remnant population of harlequin toads in northern Venezuela. The dots correspond to sampling sessions for which abundance and recruitment were estimable.

According to the ‘best demographic model’, capture probabilities varied substantially with seasons; the chances of detecting an adult within the transect were 2.3 times greater during the dry season than during the wet season. Also, the apparent survival of reproductive toads varied with sex; males had 1.24 greater chance of surviving than females. The number of recruiting reproductive adults varied with time usually increasing during the second half of the year (Fig 4). During 2007 —2012, the estimated abundance of reproductive adults varied between 24 and 89, with no increasing or decreasing trend (*F* = 0.397; *df* = 1, 20; *p* = 0.5358) (Fig 4). However, abundance increased abruptly during February 2013 due to a high peak in recruitment in the previous month (Fig 4). The total number of reproductive toads ever present in our site from 2007 —2013, or the super-population size, was estimated to be 650 females (95% CI: 580 —728) and 611 males (95% CI: 513–728) which suggests an almost equal sex ratio (1:1.06).

### Infection rates and *Bd*-induced mortality

**Fig 5:**
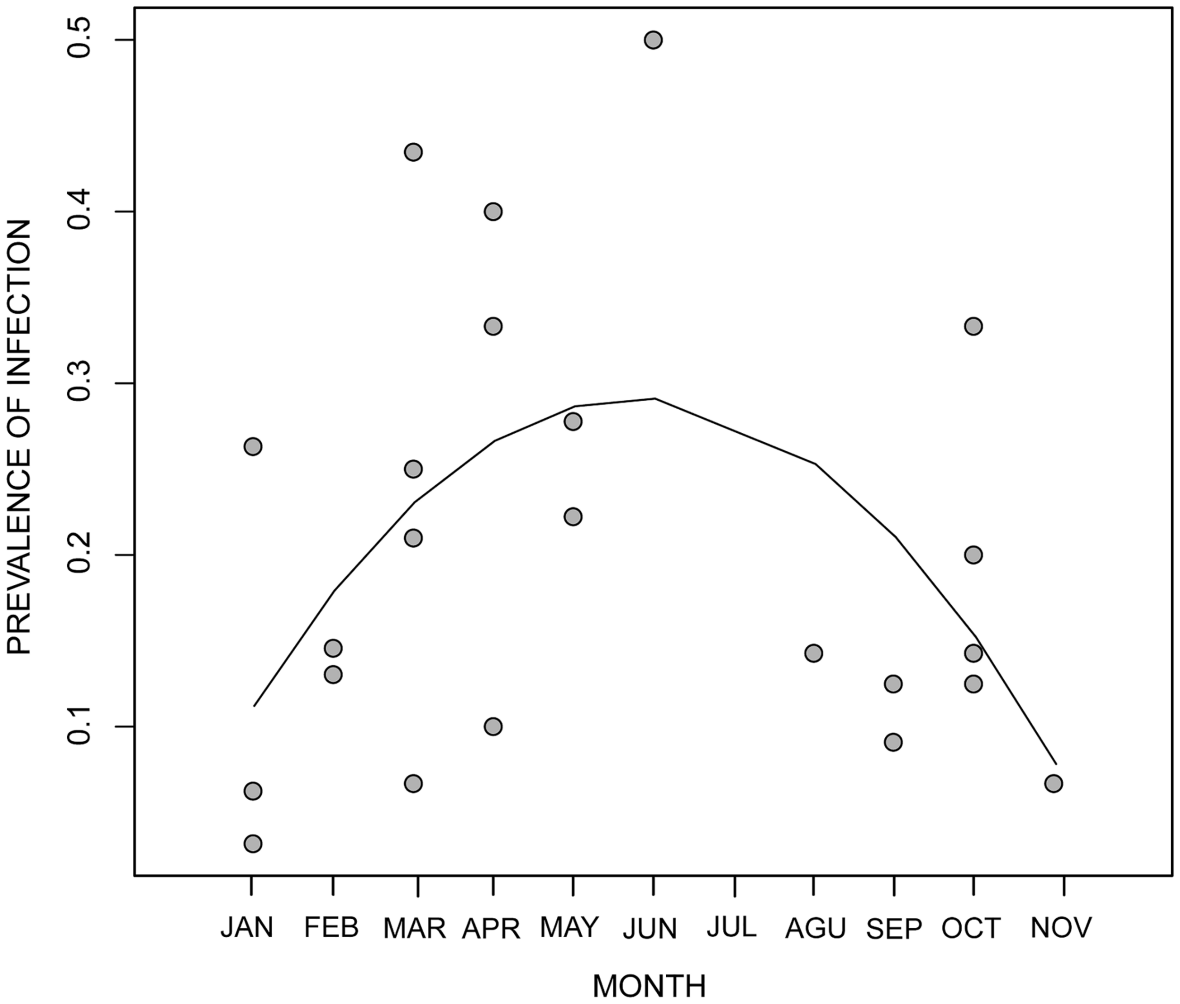
Seasonal variation in the prevalence of infection. Monthly changes in the prevalence of infection of post-metamorphic harlequin toads. The line corresponds to the second-order polynomial *Y* = *A*+B*x+C*x^2^ where *A* = 0.0295, *B* = 0.0908 and *C* = −0.00785. *R*^2^ = 0.2519. The maximum prevalence is predicted at the end of May.

**Fig 6:**
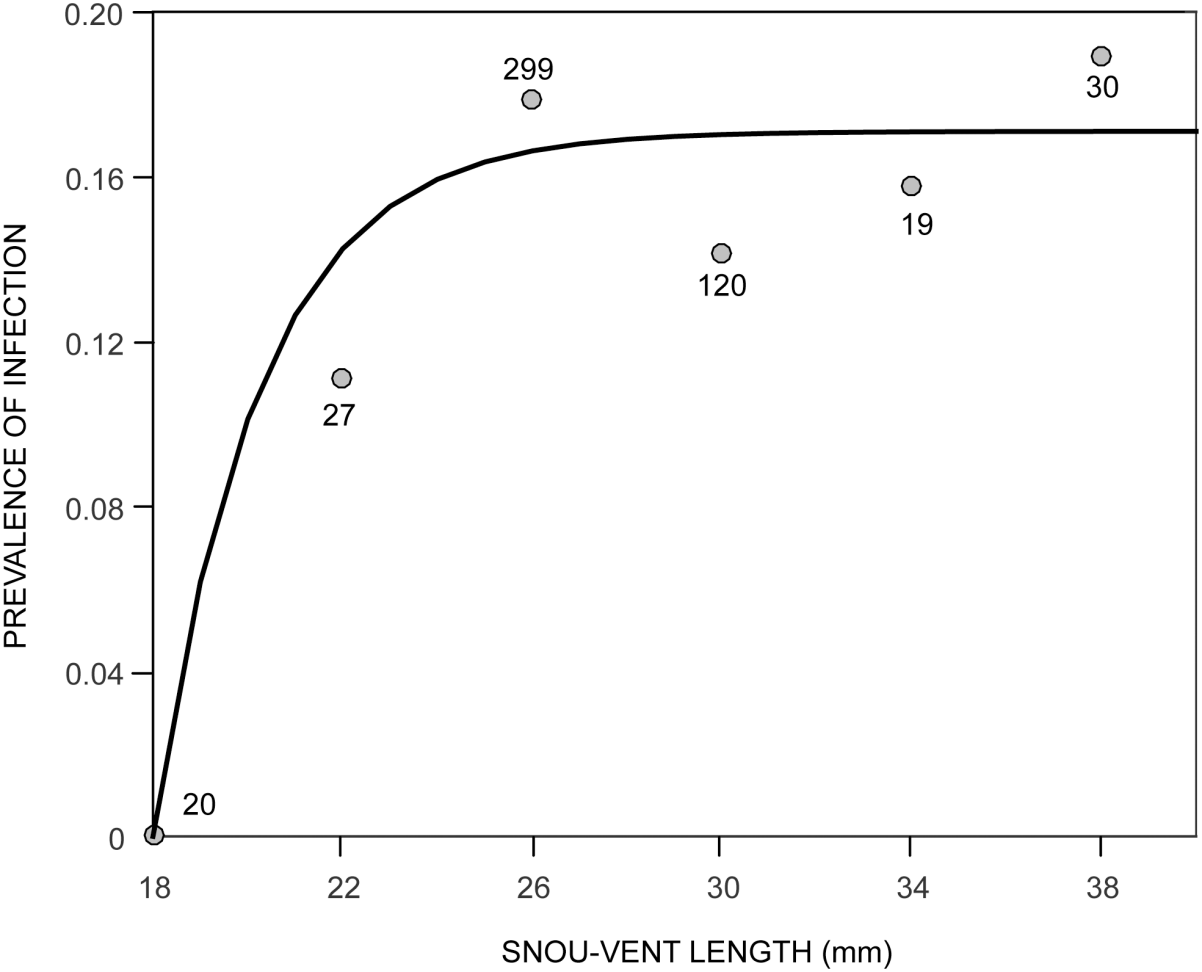
Size related changes in the prevalence of infection. Prevalence of infection of post-metamorphic toads of *A. cruciger* in six size-classes defined in the text. The curve corresponds to the best fitted function (Eq 2) with *γ* = 0.448, *S_o_* = 18 and *A* = 0.171. The 95% confidence interval for *A* indicates that an asymptotic prevalence of infection lower than one.

Sixty-seven toads were found infected (16.38%). Recapture rates varied substantially between infected and uninfected toads. Thirty percent of uninfected toads were recaptured at least once, but only 1.5% infected toads (four individuals) were subsequently recaptured. The prevalence of infection varied monthly between 3% and 50% (Fig 5). A significant reduction in the model deviance that resulted from the addition of the non-linear term indicated a maximum value in the prevalence of infection at the end of May, shortly after the dry season has finished (reproductive peak) and the number of toads near the water have began to decline (*F* = 6.618; *df* = 1; *p* = 0.018) (Fig 5). The prevalence-age relationship suggest that in post-metamorphic toads the prevalence of infection increases until it reaches a asymptotic value of 17%. The estimate of *S_o_* indicates that toads start acquiring infection when they reach an approximate size of 18 mm, and the *per capita* rate of infection, λ, is approximately 7.6% of toads for each 1-mm increment in size (Fig 6). Assuming that males grow at an approximate rate of 0.208mm/month between their first and third year of age (see Fig 3), the average infection rate of males is about 0.016/month or 0.176/year [1-(1-Ψ^UI^)^12^]. Infection intensities also varied substantially with a maximum of 651,250 genome equivalents, although we detected no seasonal pattern. All infected toads had zoospore loads less than 10^3^ except for four individuals that showed signs of morbidity, *i.e.* reddish skin, skinny complexion and slow reaction to disturbance. We found one dead female with 984,200 genome-equivalents. Only two individuals cleared the infection during the study. They showed infection intensities of 6.6 and 7.3 genome equivalents in their initial capture and tested negative subsequently.

The GOF of the saturated epidemiological model revealed no evidence of transience of newly marked toads (*χ*^2^ = 29.191; *df* = 40; *p* = 0.897) or trap-dependence of recaptured males (*χ*^2^ = 4.334; *df* = 2; *p* = 0.115). The overall GOF indicated no significant departure from the JollyMove model (*χ*^2^ = 33.525; *df* = 40; *p* = 0.822). The variance inflation factor was estimated to be 0.83 indicating no over or underdispersion of data, therefore, no correction was made for the inflation factor [46]. From the 36 models tested, the first seven accounted for 96.5% of the evidence weight (Table 2). The five top-ranking models included status- and sex-dependent survival and status-dependent detection probabilities. One of these models also included year-dependent survival (model 4) and five also had season or sex dependent detection probabilities (models 1, 2, 3 and 5). Because these five models differed little in their Akaike values (<3 units), we chose the model with the lowest number of parameters (Φ[stratum, sex], *p*[stratum], Ψ[stratum]) as the ‘best epidemiological model’. This model estimated six parameters; three survival probabilities, one capture probability and one state-transition probability. The threshold conditioned *S*-vector did not detected non-estimable parameters.

**Table 2:** Model ranking based on a small sample Akaike Information Criteria of the Jolly-Move models formulated with Multi-strata using RMark.

Models	Parameters	AICc	Delta AICc	Weight	Deviance
Φ(stratum, sex), *p*(stratum, season), Ψ(stratum)	7	878.6	0	0.326	401.0
Φ(stratum, sex), *p*(stratum, season, sex), Ψ(stratum)	8	880.1	0.511	0.153	400.5
Φ(stratum, sex), *p*(stratum), Ψ(stratum)	6	880.1	1.532	0.151	404.6
Φ(stratum, sex, year), *p*(stratum, season),Ψ(stratum)	8	880.5	1.955	0.123	400.9
Φ(stratum, sex), *p*(stratum, sex),Ψ(stratum)	7	880.9	2.362	0.100	403.4
Φ(stratum, sex, year), *p*(stratum, sex, season),Ψ(stratum)	7	882.0	3.461	0.058	400.3
Φ(stratum, sex, year), *p*(stratum),Ψ(stratum)	7	882.2	3.591	0.054	404.6

Only the eight top-ranking models are shown. For all others the model weight< 0.001.

**Fig 7:**
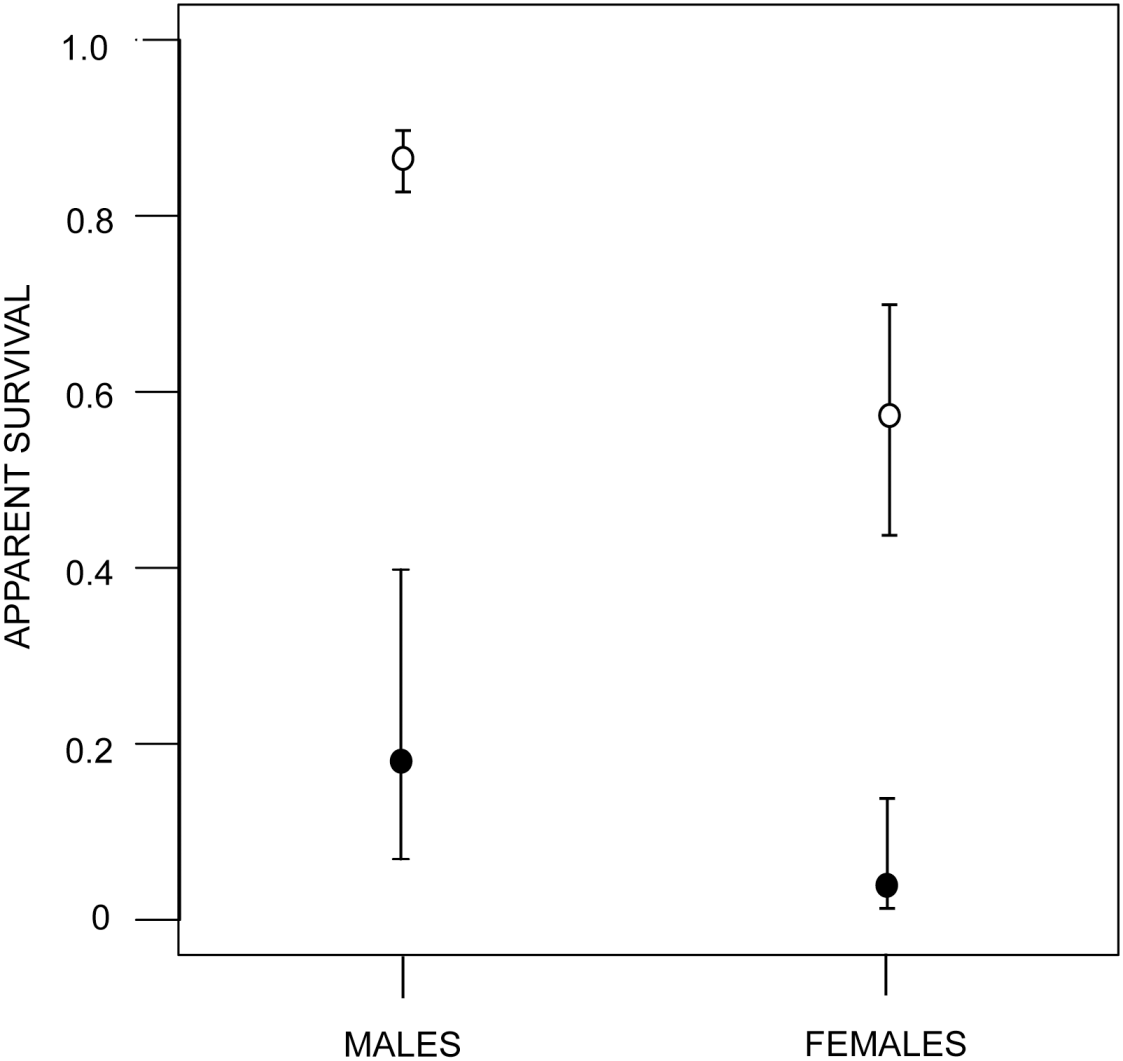
*Bd* effect on adult survival. Monthly apparent survival of reproductive toads of *A. cruciger*. White circles correspond to uninfected individuals and black circles to infected individuals. Bars indicate 95% confidence intervals.

According to the ‘best epidemiological model’, the infection status of a toad affects its detection probability. Infected toads have a four times greater chance of being detected than an uninfected individuals. This means that infected toads have a lower recapture rate as a result of their high mortality and not because a lower detection probability. This is consistent with the recurrent observations of morbid adults in the field. The MLE estimates indicated that the probability of detecting a toad was 0.244 (95% CI: 0.194–0.302), if uninfected, and 1.0 (95% CI: 0.999–1), if infected. Re-running the model from several initial values indicates that the estimate of 1.0 for the detection probability of infected toads, although at a boundary, corresponds to a global maximum [47]. Males have higher survival than females, but the presence of infection increases the odds of dying by 6.1 in males and 2.5 in females (Fig 7). According to this model, uninfected males and females have life expectancies (-1/log(Φ) of 6.9 and 1.7 months respectively. In contrast, infected males and females would die in 0.31 and 0.58 months, respectively. The annual loss due to chytridiomycosis estimated from the infection rate and the *Bd*-induced mortality was approximately 17.6% of the reproductive population.

## Discussion

Few populations of harlequin toads have persisted for decades with endemic infection with *Bd* [17, 48–51], despite the devastating effect this pathogen had in the past on most species in this genus [8, 52]. A lower *Bd* virulence in warm habitats, where it tends grow slowly, has been proposed to explain the higher survival of lowland populations of some frog species (*i.e.* thermal refuges hypothesis) [12, 53–57]. Our study demonstrates, however, that *A. cruciger* is still highly vulnerable to chytridiomycosis, despite the high ambient temperatures where this species currently survives. Survival estimates suggest that in the wild infected toads are, in average, four times more likely of dying than non-infected individuals. The life expectancy of infected toads tends to be less than a month from the detection of infection in the wild. Thus, chytridiomycosis continues to be, after three decades from its appearance, a highly lethal disease to *A. cruciger*, even in lowland warm habitats.

Despite an estimated annual loss of 18% of the reproductive population induced by chytridiomycosis, the *A. cruciger* population at Cata river have been stable at least since 2005. Although we cannot rule out emigration as a possible cause of population losses, the tendency of harlequin toads to return to the same sections within the transect year after year and the absence of nearby streams suggest that permanent emigration must be infrequent. A previous study suggested that adult toads at this location had high mortality rates but these losses were compensated by the rapid recruitment of new adults [17]. The estimated total number of adult toads and new recruits indicate an almost complete turnover of the reproductive population every year at the study site, a demographic process also observed in other frog species infected with *Bd* [58–60]. For adult recruitment to be high, however, metamorphs and juveniles must show some resistance to chytridiomycosis or have a low exposure to infection.

Early stages of *A. cruciger* are almost unknown. Tadpoles raised in captivity have been described [61], but never seen in the wild. Also, newly metamorphosed toadlets have only been sighted few times near the water because most tend to disappear from the shore and remain concealed in the forest. Extrapolation of the age-prevalence curve, however, suggests that the prevalence of infection rises rapidly from zero to its maximum value between 18 and 24 mm of length (1–1.5 years old), when toads begin to congregate along the river to mate. This indicates that *Bd* infection occurs mostly near the water and toads are first exposed to the fungus as they recruit into the reproductive population. A low exposure to infection among juveniles has also been observed in other frog species that disperse away from the water upon metamorphosis [58]. Thus, the use of habitats separated from the river’s main course during pre-adult stages appears to assure a high recruitment of healthy reproductive harlequin toads.

Life expectancy of *A. cruciger* appears to be short. Most adults do not survive to a second breeding season, and those that acquire infection probably do not breed at all because their life expectancy is reduced to less than one month. Experimentally infected toads of *A. zeteki* have also been shown to succumb to chytridiomycosis within days of exposure [62–64]. Thus, *Bd*’s major impact on harlequin toad populations would be on reproductive individuals, an effect recently evidenced by differences in the age-structure of pre- and post-infected populations of tree frogs in Australia [58, 59].

In light of the high susceptibility of *A. cruciger* to *Bd*, even in lowland habitats, the thermal refuge hypothesis needs to be redefined in terms of habitats of reduced transmission rather than diminished virulence. Given that infected toads in warm habitats survive only for few weeks, a further reduction in their life expectancy in cooler habitats is likely to have little impact on the probability of breeding and, consequently, on the host population dynamics. If high temperatures, however, significantly reduce the zoospore survival outside its host, transmission would also be diminished. Theoretical models have demonstrated that the survival of free zoospores is one of the parameters with the greatest effect on the probability of extinction of frog populations [65, 66]. This means that the observed contraction of the geographic distribution of *A. cruciger* to low altitudes could be associated to a diminished survival of free zoospores rather than better resistance to the disease.

Other studies have used the term thermal refuge to refer to habitats with lower prevalence of infection, although the underlying mechanisms leading to lower prevalence have not been explicitly defined in most cases [48, 67]. Classic epidemiological models have demonstrated that pathogen transmission rates and disease-induced mortality may both affect the prevalence of infection [41]. If we can rule out a high survival of infected toads in thermal refuges, we are only left with a reduce transmission of *Bd*. Transmission includes all steps between the release of zoospores by infected toads to the acquisition of these zoospores by susceptible toads. It seems unlikely that release rates would be low at this warm habitat because, despite the high ambient temperatures, infected toads appear to achieve lethal infection intensities (10^4^–10^6^ zoospores per swab [62]) within days of infection, and the rate of zoospore shedding by a host is proportional to the intensity of infection [62, 64]. Alternatively, a reduced survival of the free zoospore in warm habitats would lead to a lower *Bd* transmission, as we hypothesized for *A. cruciger*, because zoospore exposure of susceptible toads would also be lower. Although the distribution of zoospores in the environment is largely unknown, the number of zoospores found in water appears to be diminished at lower elevations [68]. We need a better understanding of the mechanisms affecting the transmission of *Bd* in the field, in order to asses the importance of thermal refuges for the conservation of amphibians threatened by chytridiomycosis.

## Conclusion

Understanding the host-parasite demography in remnant populations of species threatened by diseases is basic for designing their conservation strategies. Most ongoing *Bd* mitigation programs have focused on the development of prophylactic treatments on the hosts [69]. However, if the survival of the free zoospore has greater effect in the transmission rate than the host burden, targeting the zoospore outside its host body would be a more effective strategy to reduce the incidence of the disease. Chemical treatment of water, physical alteration of the microhabitat and biocontrol of zoospores using microcrustaceans have been proposed to reduce *Bd* transmission rates [69], but the distribution of free living zoospores in the habitat is mostly unknown. In addition to water, free zoospores have also been detected in leaves where frogs have recently perched [70]. Thus, we need to identify where free zoospores of *Bd* are commonly shed before we can design habitat-management strategies.

## Supporting information

S1 Dataset Capture histories of post-metamorphic individuals of the harlequin toad *Atelopus cruciger* between 2007–2013. (CSV)

S2 Dataset Infection histories of post-metamorphic individuals of the harlequin toad *Atelopus cruciger* between 2007–2013. (CSV)

S3 Dataset Monthly rainfall data obtained from weather stations at Ocumare De La Costa (2007–2011) and Maracay (2012–2013). (CSV)
